# *Naja naja karachiensis* Envenomation: Biochemical Parameters for Cardiac, Liver, and Renal Damage along with Their Neutralization by Medicinal Plants

**DOI:** 10.1155/2014/970540

**Published:** 2014-04-27

**Authors:** Muhammad Hassham Hassan Bin Asad, Ghulam Murtaza, Muhammad Ubaid, Durr-e-Sabih, Ashif Sajjad, Rubada Mehmood, Qaisar Mahmood, Muhammad Muzzmil Ansari, Sabiha Karim, Zahid Mehmood, Izhar Hussain

**Affiliations:** ^1^Department of Pharmacy, COMSATS Institute of Information Technology, Abbottabad 22060, Pakistan; ^2^Multan Institute of Nuclear Medicine and Radiotherapy (MINAR), 377, Nishtar Hospital, Multan 60000, Pakistan; ^3^Institute of Biochemistry, University of Balochistan, Quetta 87300, Pakistan; ^4^Department of Environmental Sciences, COMSATS Institute of Information Technology, Abbottabad 22060, Pakistan; ^5^Roba-al-Safwa Pharmacy, Alsafwa Hospital 67, Makkah, Saudi Arabia; ^6^University College of Pharmacy, University of the Punjab, Lahore 54000, Pakistan

## Abstract

*Naja naja karachiensis* envenomation was found to hit more drastically heart, liver, and kidneys. 400 **μ**g/kg of venom-raised moderate serum levels of ALT (72 ± 4.70 U/L, 0.1 > *P* > 0.05), AST (157 ± 24.24 U/L, 0.1 > *P* > 0.05), urea (42 ± 3.08 mg/dL, 0.05 > *P* > 0.02), creatinine (1.74 ± 0.03 mg/dL, 0.01 > *P* > 0.001), CK-MB (21 ± 1.5 U/L, 0.05 > *P* > 0.02), and LDH (2064 ± 15.98 U/L, *P* < 0.001) were injected in experimental rabbits. However, lethality was enhanced with 800 **μ**g/kg of venom in terms of significant release of ALT (86 ± 5.0 U/L, 0.05 > *P* > 0.02), AST (251 ± 18.2 U/L, 0.01 > *P* > 0.001), urea (57.6 ± 3.84 mg/dL, 0.02 > *P* > 0.01), creatinine (2.1 ± 0.10 mg/dL, 0.02 > *P* > 0.01), CK-MB (77 ± 11.22 U/L, 0.05 > *P* > 0.02), and LDH (2562 ± 25.14 U/L, *P* ≪ 0.001). Among twenty-eight tested medicinal plant extracts, only *Stenolobium stans* (L.) Seem was found the best antivenom (*P* > 0.5) compared to the efficacy of standard antidote (ALT = 52.5 ± 3.51 U/L, AST = 69.5 ± 18.55 U/L, urea = 31.5 ± 0.50 mg/dL, creatinine = 1.08 ± 0.02 mg/dL, CK-MB = 09 ± 0.85 U/L, and LDH = 763 ± 6.01 U/L). Other plant extracts were proved less beneficial and partly neutralized the toxicities posed by cobra venom. However, it is essential in future to isolate and characterize bioactive compound(s) from *Stenolobium stans* (L.) Seem extract to overcome the complications of snake bite.

## 1. Introduction

One of the animals which represent Pakistan and India throughout the world is cobra; in particular it is rearing out of a wicker basket and dancing to the sound of turban-wearing snake charmer music. In fact, they are deadly venomous among all species of the snakes due to high rate of mortality which strongly reasoned for their notoriety [1]. At the end of the nineteenth century, all cobra populations have been merged into single species naja (formerly known as naia); however, subsequently ten subspecies of* Naja naja* were identified [1]. Later on, Deraniyagala classified* Naja naja* (India) into several subspecies and considered* Naja naja karachiensis* one of them widely distributed in Southern Punjab province of Pakistan [2]. They are extremely toxic due to their severe side effects in the victims. Major signs and symptoms of cobra envenomation are edema, necrosis, pain, respiratory paralysis, vomiting, headache, hypotension, cardiac arrest, coagulopathies (elevated PT, aPTT, and TT), bleeding wounds, hematuria, mucus discharge, proteinuria, and increased creatinine and urea levels along with altered consciousness [3, 4]. 

Indeed snake venom is a complex mixture of various sizes of polypeptides [5, 6] (<1.5 kDa, 5 to 10 kDa, and 10 to 150 kDa), carbohydrates, lipids, metal ions, organic molecules, purines, and amines [7, 8]. Snake bite envenomation has been attributed to pose drastic changes in the physiology of the victims that could be accessed clinically by measuring various biochemical parameters. Among toxic components, phospholipases A_2_ (cell destroying enzymes cause edema, lipolytic or myolytic) [9, 10], phosphodiesterases (nucleic acid degrading enzyme causes hypotension/shock) [11, 12], 5′ nucleotidases (digest the sugar moities causing the delay in coagulation) [13], alkaline phosphatases (hydrolyze phosphate esters nonspecifically cause multiple toxicities via adenosine generation) [14], hyaluronidases (spreading factor causes digestion, necrosis, and leakage of blood vessels) [15], L-amino oxidases (deamination leads to cell damage/apoptosis) [16], and proteases (hydrolyze various proteins) [17] are included.

Laboratory animals, particularly rabbits, have been selected previously to monitor various biochemical changes related to liver, heart, and renal toxicities [18, 19]. Aspartate aminotransferase (GOT/AST) and alanine aminotransferase (GPT/ALT) are surrogate markers for liver toxicities while creatine kinase isoenzyme (CK-MB) is used exclusively to access cardiac tissue damage [20, 21]. Elevated creatinine and urea levels are indicators of 75% renal damage [20], whereas high level of lactate dehydrogenase (LDH) implies general toxicity related to the liver, heart, kidneys, and skeleton muscles [22].

Diverse immunological properties of different cobras have fascinated protein chemists and other researchers to carry out efforts for the development of new antidotes. Due to this reason, for the first time, venom from* Naja naja karachiensis* is selected to evaluate its toxicity* in vivo* and to search out effective as well as cheap treatment from folk herbal remedies. Pakistan is a hub of medicinal plants where mostly rural communities rely on natural herbs to treat their problems of snake bite [6, 23]. It is therefore necessary to evaluate scientifically their folklore claims as anti-snake venom in traditional system of medicine. Due to this reason, various medicinal plants (*Albizia lebbeck *(L.) Benth.,* Allium cepa *L.,* Allium sativum* L.,* Althaea officinalis* L.,* Bauhinia variegata *L.,* Brassica nigra *(L.) W. D. J. Koch*, Calotropis procera *(Aiton) W. T. Aiton,* Cedrus deodara *(Roxb. ex D. Don) G. Don,* Citrullus colocynthis *(L.) Schrad*, Citrus limon *(L.) Burm. f.*, Cuminum cyminum *L.,* Enicostema hyssopifolium *(Willd.) I. Verd,* Fagonia cretica *L.,* Leucas capitata *Desf.,* Matthiola incana *(L.) W. T. Aiton,* Momordica charantia *L.,* Nerium indicum *Mill,* Ocimum sanctum* L.*, Pinus roxburghii *Sarg,* Pistacia integerrima *J. L. Stewart*, Psoralea corylifolia *L.,* Rhazya stricta *Decne,* Rubia cordifolia *L*., Sapindus mukorossi *Gaertn,* Solanum xanthocarpum* Schard and Wendle,* Stenolobium stans *(L.) Seem,* Terminalia arjuna *(Roxb. ex DC.) Wight and Arn,* Trichodesma indicum *(L.) Sm, and* Zingiber officinale *Roscoe) were collected to test their potentials against toxicities induced by* Naja naja karachiensis* envenomation (Table 1).

## 2. Material and Methods

### 2.1. Collection and Milking of Snake Venom

Black Pakistani cobra snakes (*Naja naja karachiensis*) were collected from Cholistan desert located in southern Punjab province of Pakistan. After collection, they were properly identified by a zoologist. Venom from* Naja naja karachiensis* was collected by pressing their glands below their eyes in low light environment. After collection, it was lyophilized and stored in light resistant container at 2°C to 8°C. For further experiments, venom was used in terms of its dry weight [6].

### 2.2. Collection and Preparation of Plant Extracts

Medicinal plants were collected from different areas in Pakistan. After collection, they were duly identified by expert botanist (Professor Dr. Altaf Ahmad Dasti) and voucher specimens were deposited in the herbarium of the Institute of Pure and Applied Biology, Bahauddin Zakariya University, Multan, Pakistan. Complete description about evaluated medicinal plants is summarized in Table 1. One kilogram chopped plant material was soaked in 5 L of methanol as solvent in extraction bottles. The homogenates were kept for a period of a month at optimum temperature. After filtration, filtrate was evaporated by using water bath and extracts were weighed and stored for further experimentation [24].

### 2.3. Experimental Animals

Seventy-one healthy growing male rabbits (1 ± 0.5 kg) were selected for this study after getting permission from local ethical committee. Animals were acclimatized for a week in the laboratory by maintaining their standards for chow, water, and light. Subsequently, they were divided into various groups in different cages such that experimental rabbits in group I were used for baseline measurements of various biochemical parameters. Animals belonging to groups II and III were evaluated for different doses of cobra venom while group IV was served as control. Group V was redivided into twenty-nine groups such that each subgroup (V/1–V/29) was assigned to a single medicinal plant extract [25].

### 2.4. *In Vivo* Antivenom Activity of Medicinal Plant Extracts

Venom from* Naja naja karachiensis* (400 *μ*g/kg and 800 *μ*g/kg) was injected subcutaneously to evaluate its toxicity to the heart, liver, and kidneys. Before injection of venom, experimental animals were anaesthetized by administration of ketamine (50 mg/kg). To determine antivenom activity of various plants' extract, they (100 mg/kg) were incubated (at 37°C for 30 minutes) with fixed amount of venom (800 *μ*g/kg) before injection while saline was used as control [26–28].

### 2.5. Biochemical Assays

For serum analysis of various biochemical parameters, blood was collected from marginal ear artery by the use of hypodermic syringe needle after 3 hours of envenomation [25]. Separated serum was tested for alanine aminotransferase (GPT/ALT), aspartate aminotransferase (GOT/AST), urea, creatinine, creatine kinase isoenzyme MB (CK-MB), and lactate dehydrogenase (LDH) by the use of kits (manufactured by Merck) according to the DGKC and IFCC method on the Selectra Junior (Vital Scientific B.V, The Netherlands) [26].

### 2.6. Statistical Analysis

All numerical values were expressed as mean (3 replicates) ± standard error of mean (SEM). They were calculated by the use of Microsoft Excel 2007 and Student's* t*-test was applied to compare the efficacy of evaluated samples with standard antidote (reference standard).

## 3. Results

Venom from* Naja naja karachiensis* was proved to cause severe complications in dose-dependent manner. Venom at the dose of 400 *μ*g/kg led to release of moderate serum levels of ALT (72 ± 4.70 U/L, 0.1 > *P* > 0.05), AST (157 ± 24.24 U/L, 0.1 > *P* > 0.05), urea (42 ± 3.08 mg/dL, 0.05 > *P* > 0.02), creatinine (1.74 ± 0.03 mg/dL, 0.01 > *P* > 0.001), CK-MB (21 ± 1.5 U/L, 0.05 > *P* > 0.02), and LDH (2064 ± 15.98 U/L, *P* < 0.001) and thus indicated toxicities to the heart, liver, and kidneys. At 800 *μ*g/kg of cobra venom, severe tissue damage was observed in terms of significant release of ALT (86 ± 5.0 U/L, 0.05 > *P* > 0.02), AST (251 ± 18.2 U/L, 0.01 > *P* > 0.001), urea (57.6 ± 3.84 mg/dL, 0.02 > *P* > 0.01), creatinine (2.1 ± 0.10 mg/dL, 0.02 > *P* > 0.01), CK-MB (77 ± 11.22 U/L, 0.05 > *P* > 0.02), and LDH (2562 ± 25.14 U/L, *P* ≪ 0.001). Complete detail about various biochemical parameters for baseline measurements different doses of cobra venom, and saline (as negative control) is summarized in Table 2.

**Figure 1:**
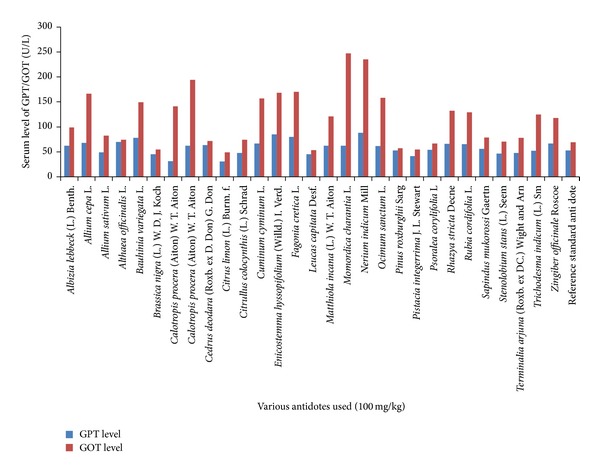
Comparison of various medicinal plant extracts with reference standard antidote in neutralization of increased GPT and GOT serum level posed by* Naja naja karachiensis* venom in rabbits.

**Figure 2:**
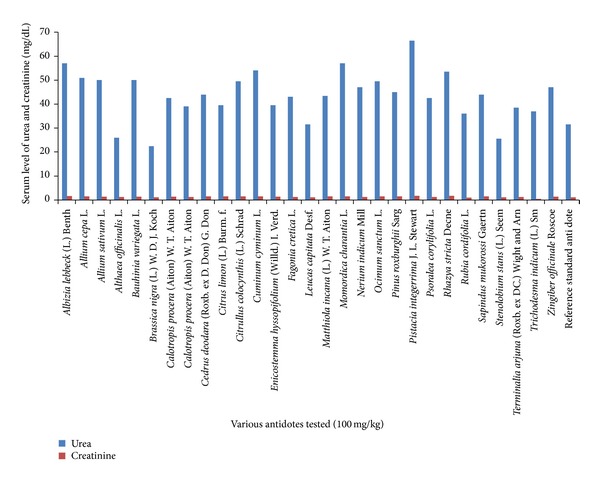
Comparison of various medicinal plant extracts with reference standard antivenom in neutralization of elevated serum urea and creatinine level posed by* Naja naja karachiensis* venom in rabbits.

**Figure 3:**
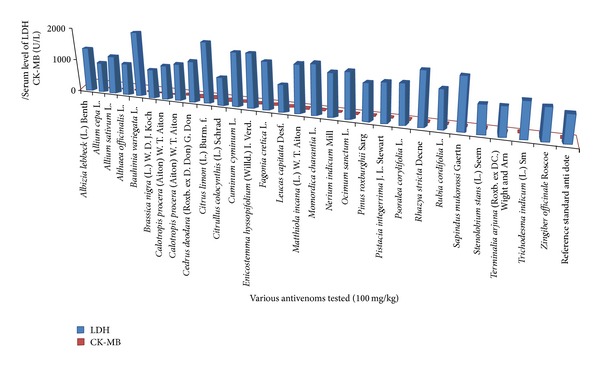
Comparison of various medicinal plant extracts with reference standard antisera in neutralization of LDH and CK-MB levels elevated by* Naja naja karachiensis* venom in rabbits.

To neutralize* in vivo* 800 *μ*g/kg of cobra venom, twenty-eight medicinal plant extracts were evaluated. It was noticed that extract of* Stenolobium stans* (L.) Seem was the best antidote (*P* > 0.5) compared to reference standard.* Allium sativum* L.,* Althaea officinalis *L.,* Citrullus colocynthis *(L.) Schrad,* Leucas capitata *Desf.,* Pinus roxburghii *Sarg,* Psoralea corylifolia* L.,* Rubia cordifolia* L., and* Sapindus mukorossi *Gaertn were found to be valuable plants to protect liver damage (*P* > 0.5) as standard antidote (ALT = 52.5 ± 3.51 U/L and AST = 69.5 ± 18.55 U/L); however, required ALT and AST levels were not observed with remaining plant extracts (0.5 > *P* > 0.05) as shown in Table 3 and comparison is shown in Figure 1. Extracts of* Leucas capitata *Desf. and* Althaea officinalis *L. were observed to be helpful (*P* > 0.5) as reference standard (urea = 31.5 ± 0.50 mg/dL and creatinine = 1.08 ± 0.02 mg/dL); nevertheless, remaining plants were found less valuable (0.5 > *P* > 0.01) to minimize urea and creatinine levels to maintain kidney functions (Table 4 and Figure 2). When compared with standard antidote (CK-MB = 9.0 ± 0.85 U/L) eight medicinal plants (*Allium cepa *L.,* Althaea officinalis *L.,* Bauhinia variegata *L.,* Cedrus deodara *(Roxb. ex D. Don) G. Don,* Fagonia cretica* L.,* Leucas capitata *Desf.*, Momordica charantia* L., and* Ocimum sanctum* L.) were found equally capable (*P* > 0.5) of normalizing high values of CK-MB; however, rest of all were proved less beneficial (0.5 > *P* > 0.1). To combat highly raised values of LDH,* Althaea officinalis *L.,* Leucas capitata *Desf., and* Terminalia arjuna* (Roxb. ex DC.) Wight and Arn were proved useful to some extent (0.5 > *P* > 0.1) although remaining plant extracts could not be shortlisted (0.1 > *P* > 0.001) as standard antisera (LDH = 763 ± 6.01 U/L). Overall detail about LDH is discussed in Table 5 and comparison is shown in Figure 3.

## 4. Discussion

Snake bite has been responsible for tens of thousands of deaths worldwide and numerous physical handicaps [29]. Generally, snake venom is an intricate mixture of various proteins (>90%) and most of them are enzymes, particularly (40%) phospholipases A_2_ [30]. Among different complications, hepatic injury is one of the deadly venomous effects produced by cobra bite [31, 32].* Naja naja karachiensis* venom caused significant increase in ALT and AST levels (dose dependently) that are surrogate markers for liver toxicity either by direct action or immunological (hypersensitivity) reaction. The allergic reactions reactions are not dose dependent which clarify lethal effects to the cytoplasm and mitochondrial membranes of the hepatic cells [25, 33]. Phospholipases enzymes, abundant in* Naja naja karachiensis* venom, are responsible for breakdown of membranous phospholipids and therefore resulted in cellular injury along with inflammation [24]. PLA_2_ is attributed to decrease in Na^+^/K^+^ ATPase activities and led to greater influx of sodium ions and water molecules into the cell. Subsequently, plasma membrane lipid bilayer disorganized and eventually resulted in hepatic cells death [33, 34]. In addition, PLA_2_ is also found to cause anticoagulation in victims [6]. All pharmacological effects are due to phospholipid hydrolysis or phospholipid competing biding mechanism with other coagulation factors, particularly FXa, or by both ways; however, it is very hard to pinpoint exact one [35]. Anticoagulant response posed by cobra PLA_2_ (anticoagulant enzyme) may aggravate hepatic injury as many anticoagulant agents cause liver damage that are idiosyncratic in nature [36].

Cardiac injury, particularly systolic heart arrest, is one of the well-known toxicities related to* Naja naja* subspecies [30]. In present study, Pakistani cobra venom was found to release two cystolic enzymes (LDH and CK-MB) that are sensitive indicators of myocyte injury [37]. It is due to the presence of myotoxic PLA_2_ and other cardiotoxin(s) that are salient features of cobra venom that are responsible for cellular necrosis and cytotoxicity [38, 39]. Cytotoxic effect of* Naja naja karachiensis* venom may not be overlooked even when antisera administration is late cause of toxic components to the microvasculature and thrombus produced which poses hindrance in access of immunoglobulins to the site of snake bite [26, 29, 39].

Snake venom has been responsible for detrimental effects to the renal tissues [40]. Likewise,* Naja naja krachiensis *venom was found to cause severe renal damage by significant rise in serum urea and creatinine levels. Indeed, phospholipases enzymes are responsible for the increase in vascular permeability with hemorrhagic effects to the vital tissues in the victims. Subsequently, numerous lesions are produced related to the glomerular membrane and renal tubules either interstitial or vascular [29, 41, 42]. Presence of lymphocytes (white blood cells) and oedema in the cortical as well as medullary regions of renal tubules further confirmed the idea of renal damage (data has not shown) as reported previously with different snake venoms like* Hemiscorpius lepturus* [26, 43]. Apart of it, pharmacokinetic studies of* Naja naja karachiensis* venom with short lived radioisotope ^99m^Tc confirmed that kidneys and urinary bladder are the most saturated organs (>70%) after intravenous injection in experimental rabbits (unpublished data by our group).

Medicinal plants of Pakistan are used to inhibit snake venom (PLA_2_) enzymes [6]. Due to this effect, present study was designed to select twenty-eight medicinal plants of Pakistan to test their potentials as an antidote against toxicities produced by* Naja naja karachiensis* venom. It was fascinating that extract of* Stenolobium stans* (L.) Seem showed significant neutralization compared to that of reference standard (antisera) as reported previously by Asad et al., for inhibition of phospholipases A_2_ anticoagulant activity [6, 44]. Present study indicated that extract of* Stenolobium stans* (L.) Seem possesses an endogenous inhibitor(s) to nullify venom (PLA_2_) induced toxicities. Extracts of these plants material are routinely used in Pakistan by simple application in the form of paste to the affected area [45]. This practice provides effective first aid treatment as tiny molecules of an antidote diffuse favorably at the site of snake bite before hospitalization and abrogated the spreading of toxins [39]. It is owing to the various secondary metabolites like phenols, flavonoids, terpenoids, xanthenes, quinonoids, and so forth, as reported earlier to mask various enzymatic actions of cobra venom [6, 23]. Indeed, secondary metabolites pose hindrance in binding of different snake venom enzymes to their potential targets; therefore, antidotal effect evoked. Other plants extract were not proved significantly effective to neutralize cobra venom, therefore cannot be declared useful plants in venom therapy. It is the need of the time to isolate lethal component(s) of cobra venom attributed to its major toxicities. Furthermore, characterization of antivenom compound(s) from medicinal plant extracts would be worth full for complete and effective treatment of snake bite in the future.

## Figures and Tables

**Table 1:** Detailed description for evaluated medicinal plants having folklore evidences as antivenom.

Sr. number	Botanical name of medicinal plants	Family	Part used	Phytochemicals reported	Reference
1	*Albizia lebbeck* (L.) Benth.	Fabaceae	Seed	Carbohydrates, proteins, alkaloids, flavanoids, tannins, echinocystic acid, and different amino acids.	[6]

2	*Allium cepa *L.	Amaryllidaceae	Bulb	11 g carbohydrates, 1.2 g proteins, 0.6 g fibers, and H_2_O content (86.8 g)/100 g of material.	[6]

3	*Allium sativum* L.	Amaryllidaceae	Bulb	Quercetin, scordinines A and B allicin, thiosulfinates, 2 mercapto-L-cysteines, anthocyanins, alliinase, polysaccharides, sativin I and sativin II, and glycosides of kaempferol.	[6]

4	*Althaea officinalis *L.	Malvaceae	Root	37% starch, 11% mucilage, fatty oil, pectin, flavonoids, phenolic acids, asparagines, phytosterol, sucrose, and butyric acid.	[6]

5	*Bauhinia variegata* L.	Fabaceae	Root	Tannins, fatty oil, lupeol, kaempferol-3-glucoside, gums, *β*-sitosterol, 5,7-dehydroxy and 5,7-dimethoxy-flavanone-4-0-a-L-rhamnopyranosyl-*β*-D glucopyranosides.	[6]

6	*Brassica nigra* (L.) W. D. J. Koch	Brassicaceae	Seed	Essential oil, sinigrin and glucoside.	[6]

7	*Calotropis procera* (Aiton) W. T. Aiton	Apocynaceae	Exudates and flower	Resins, tannins, calotropin, sterol, uscharin, calotropagenin, calotoxin, and calactin.	[6]

8	*Cedrus deodara* (Roxb. ex D. Don) G. Don	Pinaceae	Bark	Taxifolin, dewardiol, dewarene, gum, ascorbic acid, himadarol, cantdarol, cholesterin, allohimachalol, cedrinoside, himachalol, dewarol, cedrin, dihydrodehydrodiconiferyl alcohol, essential oil, isocentdarol, and dihydromyricetin.	[6]

9	*Citrus limon* (L.) Burm. f.	Rutaceae	Fruit	4-*β*-glucopyranoside, d-x-pinene camphene, d-limonene, linalool ichangin 4-*β*-glucopyranoside, nomilinic acid, and essential oil.	[6]

10	*Citrullus colocynthis* (L.) Schrad	Cucurbitaceae	Fruit	Many alkaloids, glycosides, tannins, citrulluin, citrulluic acid, dihydric alcohol, phydroxybenzyl, citrulluene, hentriacontane, elaterin, citrullol, methyl ether, bitter oil, citbittol, and saponins.	[6]

11	*Cuminum cyminum* L.	Apiaceae	Seed	Essential oil and cumin oil contain sminaldehyde, 1,3-p-menthadien-7-al, and 1,4-p-menthadien-7-al.	[6]

12	*Enicostema hyssopifolium* (Willd.) I. Verd.	Gentianaceae	Whole plant	Saponarin, isovetexin, sylyswertin, betulin, swertiamarin, apigenin, genkwanin, erythrocentaurine, swertioside, isoswertisin, enicoflavine, and swertisin.	[6]

13	*Fagonia cretica* L.	Zygophyllaceae	Leaves	Ursolic acid, pinitol, nahagenin, saponin glycosides, saponin-I and saponin-II, docosyl docosanoate from n-hexane extract, and different proteins from aqueous extract.	[6]

14	*Leucas capitata *Desf.	Lamiaceae	Whole plant	Alkaloid as well as essential oil.	[6]

15	*Matthiola incana* (L.) W. T. Aiton	Brassicaceae	Seeds	Oil rich in chlorophylls a, b, N, P, K, and Na, *γ*-linolenic acid, and carotenoids. Sulforaphene is an important component.	[6]

16	*Momordica charantia* L.	Cucurbitaceae	Fruit	Carotenoids, momorcharasides A and B, glucosides, stigmastadien-3-beta-ol, squalene, mycoses, steroidal glucoside, momordicines I and II, stigmasterol, vicine, cucurbitane triterpenoids, momordicosides, cycloeucalenol, taraxerol, spinasterollophenol, diosgenin, thiocyanogen, 24-methylencycloartenol, and phenyl propanoids.	[6]

17	*Nerium indicum* Mill	Apocynaceae	Root and leaves	Nerioderin, neriodorin, karabin, and odorin.	[6]

18	*Ocimum sanctum* L.	Lamiaceae	Whole plant	Essential oils are found rich in 3.2% carvacrol, 71.3% eugenol, 1.7% caryophyllene, 20.4% methyl eugenol, eugenol methyl ether, linalool, and methyl chavicol cineole.	[6]

19	*Pinus roxburghii* Sarg	Pinaceae	Oleoresin	Careen, *α*-pinene, *β*-pinene, *β*-carene, *β*-longifolene, longifolene, and longicyclene.	[6]

20	*Pistacia integerrima *J. L. Stewart	Anacardiaceae	Galls	1.3% essential oil rich in camphene, cineole, A-terpineol, A-pinene, aromadendrene, caprylic acid, and d-limonene abundant in galls.	[6]

21	*Psoralea corylifolia* L.	Fabaceae	Seeds	Limonene, linalool, psoralene, angelicin, neobavachalcone, bakuchiol, 4-terpineol, corylidin, neobavaisoflavone, bavachinin, isobavachin, *α*-elemene, geranylacetate, stigmasterol, bakuchioltraincontane, isopsoralidin, isopsoralen, bakuchalcone, isoneobavachalcone, psoralen, psoralidin, raffinose, corylifolinin, corylifolean, and corylifolin.	[6]

22	*Rhazya stricta* Decne	Apocynaceae	Leaves	Enzymes (NADPH dependent), glycosides (roblnin, 3-7-rhamnoside and isorhamnetin-3-7-rhamnoside), alkaloid (sewarine), and triterpenes (ursolic acid, Mg quinate, and *β*-sitosterol) along with flavonoids rhazianosides A and B.	[6]

23	*Rubia cordifolia* L.	Rubiaceae	Stem	Pseudopurpurin, xanthopurpurin, purpurin, munjistin, alizarin, and munjistin are found with their glycosides.	[6]

24	*Sapindus mukorossi* Gaertn	Sapindaceae	Fruit	*β*-Sitosterol, sapindoside A, sapindoside B, kaempferol, quercetin, saponin, and emarginatoside.	[6]

25	Stenolobium stans (L.) Seem	Bignoniaceae	Root	*β*-Carotene, *β*-sitosterol, *α*-amarine, zeaxanthin, indole metabolizing enzymes, phenolic acids, oleanolic acid, triterpenoids, ursolic acid, indole-oxygenase, and luteinzeaxanthin.	[6]

26	*Terminalia arjuna* (Roxb. ex DC.) Wight and Arn	Combretaceae	Bark	*β*-Sitosterol, arjunolic acid, tomentosic acid, ellagic acid, essential oil, arjunetin, arjunine, calcium salts, aluminium and magnesium salts, coloring agents, reducing sugars, tannin (pyrocatechol), and a lactone.	[6]

27	*Trichodesma indicum* (L.) Sm	Boraginaceae	Whole plant	Seeds oil is rich in linoleic, oleic, stearic, palmitic, and linolenic acids. Apart of it hexacosane, ethyl hexacosanoate, and 21,24-hexacos-adienoic acid ethyl esters are found.	[6]

28	*Zingiber officinale* Roscoe	Zingiberaceae	Rhizome	D-Curcumene, cineol, potassium oxalate, camphene, citral borneol, singiberine, shogaol, *α*-curcumene, *α*-bergamotene, *β*-gamma-bisabolene, gingerol, and *β*-phellandrene.	23

**Table 2:** Biochemical parameters before and after snake venom administration in different experimental groups of rabbits.

Toxicity determined	Markers of snake venom toxicity	Group I baseline measurements (mean ± SEM)	Group II and III venom injection		Group IV negative control (mean ± SEM)	Normal reference values reported	References
			0.4 mg/kg (mean ± SEM)	0.8 mg/kg (mean ± SEM)			
Liver	GPT/ALT	(52 ± 3.46) U/L	(72 ± 4.70) U/L 0.1 > *P* > 0.05	(86 ± 5.0) U/L 0.05 > *P* > 0.02	(52 ± 3.48) U/L	(48–80) U/L	[19]
	GOT/AST	(65 ± 6.57) U/L	(157 ± 24.24) U/L 0.1 > *P* > 0.05	(251 ± 18.2) U/L 0.01 > *P* > 0.001	(67 ± 3.21) U/L	(14–113) U/L	[19]

Kidneys	Urea	( 28 ± 1.73) mg/dL	(42 ± 3.08) mg/dL 0.05 > *P* > 0.02	(57.6 ± 3.84) mg/dL 0.02 > *P* > 0.01	(28 ± 0.33) mg/dL	(10–28) mg/dL	[26]
	Creatinine	(1.0 ± 0.313) mg/dL	(1.74 ± 0.03) mg/dL 0.01 > *P* > 0.001	(2.1 ± 0.10) mg/dL 0.02 > *P* > 0.01	(1 ± 0.06) mg/dL	(0.5–2.5) mg/dL	[26]

Heart	CK-MB	(13.2 ± 2.0) U/L	(21 ± 1.5) U/L 0.05 > *P* > 0.02	(77 ± 11.22) U/L 0.05 > *P* > 0.02	(13 ± 0.56) U/L	(<25) U/L*	[20, 21]
	LDH	(714 ± 3.18) U/L	(2064 ± 15.98) U/L *P* < 0.001	(2562 ± 25.14) U/L *P* ≪ 0.001	(720 ± 4.70) U/L	(559–2077) U/L	[46]

*CK-MB activity is less than 2% in healthy rabbits; however, it is usually 10%–30% of total CK activity.

**Table 3:** Hepatoprotective activity of various medicinal plant extracts on ALT and AST levels to neutralize snake bite envenomation in experimental rabbits.

Sr. number	Group V (subgroup)	Name of evaluated sample	GPT/ALT		GOT/AST	
			U/L (mean ± SEM)	*P* value/comment	U/L (mean ± SEM)	*P* value/comment
1	(V/1)	*Albizia lebbeck *(L.) Benth.	62 ± 7.02	0.5 > *P* > 0.1	99 ± 18.55	0.5 > *P* > 0.1
2	(V/2)	*Allium cepa *L.	68 ± 12.03	0.5 > *P* > 0.1	166.5 ± 0.50	0.5 > *P* > 0.1
3	(V/3)	*Allium sativum *L.	49 ± 5.01	*P* > 0.5	82.5 ± 18.55	*P* > 0.5
4	(V/4)	*Althaea officinalis*L.	70 ± 20.05	*P* > 0.5	74.5 ± 14.54	*P* > 0.5
5	(V/5)	*Bauhinia variegata *L.	78 ± 4.04	0.5 > *P* > 0.1	149.5 ± 19.9	0.5 > *P* > 0.1
6	(V/6)	*Brassica nigra *(L.) W. D. J. Koch	45 ± 0.00	0.5 > *P* > 0.1	55 ± 1.00	*P* > 0.5
7	(V/7a)	*Calotropis procera *(Aiton) W. T. Aiton (exudates)	31.5 ± 0.5	0.5 > *P* > 0.1	141 ± 1.01	0.5 > *P* > 0.1
8	(V/7b)	*Calotropis procera *(Aiton) W. T. Aiton (flowers)	62.5 ± 2.52	0.5 > *P* > 0.1	194 ± 12.12	0.5 > *P* > 0.1
9	(V/8)	*Cedrus deodara (*Roxb. ex D. Don) G. Don	63.3 ± 4.14	0.5 > *P* > 0.1	72 ± 2.02	*P* > 0.5
10	(V/9)	*Citrus limon *(L.) Burm. f.	30.5 ± 13.63	0.5 > *P* > 0.1	49 ± 24.24	*P* > 0.5
11	(V/10)	*Citrullus colocynthis *(L.) Schrad	47.5 ± 5.55	*P* > 0.5	74 ± 9.09	*P* > 0.5
12	(V/11)	*Cuminum cyminum *L.	67 ± 16.16	*P* > 0.5	157 ± 14.14	0.5 > *P* > 0.1
13	(V/12)	*Enicostemma hyssopifolium *(Willd.) I. Verd.	85.25 ± 1.26	0.1 > *P* > 0.05	168.5 ± 7.57	0.5 > *P* > 0.1
14	(V/13)	*Fagonia cretica *L.	80 ± 7.82	0.5 > *P* > 0.1	170 ± 13.13	0.5 > *P* > 0.1
15	(V/14)	*Leucas capitata *Desf.	45.5 ± 7.52	*P* > 0.5	53.5 ± 0.50	*P* > 0.5
16	(V/15)	*Matthiola incana *(L.) W. T. Aiton	62 ± 8.08	0.5 > *P* > 0.1	121 ± 5.05	0.5 > *P* > 0.1
17	(V/16)	*Momordica charantia *L.	62.5 ± 7.52	0.5 > *P* > 0.1	247 ± 46.13	0.5 > *P* > 0.1
18	(V/17)	*Nerium indicum *Mill	88 ± 19.55	0.5 > *P* > 0.1	235 ± 18.05	0.1 > *P* > 0.05
19	(V/18)	*Ocimum sanctum *L.	61.5 ± 7.52	0.5 > *P* > 0.1	158 ± 10.02	0.5 > *P* > 0.1
20	(V/19)	*Pinus roxburghii *Sarg	53 ± 9.02	*P* > 0.5	57 ± 13.03	*P* > 0.5
21	(V/20)	*Pistacia integerrima *J. L. Stewart	41.5 ± 1.5	0.5 > *P* > 0.1	54.5 ± 0.50	*P* > 0.5
22	(V/21)	*Psoralea corylifolia *L.	54 ± 1.00	*P* > 0.5	66.5 ± 11.53	*P* > 0.5
23	(V/22)	*Rhazya stricta *Decne	66 ± 24.24	*P* > 0.5	132.5 ± 12.62	0.5 > *P* > 0.1
24	(V/23)	*Rubia cordifolia *L.	65.5 ± 17.67	*P* > 0.5	129 ± 66.41	*P* > 0.5
25	(V/24)	*Sapindus mukorossi *Gaertn	56 ± 6.01	*P* > 0.5	78.5 ± 13.54	*P* > 0.5
26	(V/25)	*Stenolobium stans *(L.) Seem	46.5 ± 6.56	*P* > 0.5	70.5 ± 3.53	*P* > 0.5
27	(V/26)	*Terminalia arjuna *(Roxb. ex DC.) Wight and Arn	47.5 ± 1.51	0.5 > *P* > 0.1	78 ± 6.06	*P* > 0.5
28	(V/27)	*Trichodesma indicum *(L.) Sm	52 ± 0.00	*P* > 0.5	125 ± 4.01	0.5 > *P* > 0.1
29	(V/28)	*Zingiber officinale *Roscoe	66.5 ± 32.59	*P* > 0.5	117.5 ± 9.52	0.5 > *P* > 0.1
30	(V/29)	Reference standard antidote (standard antisera /immunoglobulin's)	52.5 ± 3.51	Select to compare	69.5 ± 18.55	Select to compare

**Table 4:** Nephroprotective activity of different medicinal plant extracts on urea and creatinine levels to neutralize snake bite envenomation in experimental rabbits.

Sr. number	Group V (subgroup)	Name of evaluated sample	Urea		Creatinine	
			mg/dL (mean ± SEM)	*P* value/comment	mg/dL (mean ± SEM)	*P* value/comment
1	(V/1)	*Albizia lebbeck *(L.) Benth.	57 ± 0.0	0.02 > *P* > 0.01	1.6 ± 0.20	0.5 > *P* > 0.1
2	(V/2)	*Allium cepa *L.	51 ± 11.03	0.5 > *P* > 0.1	1.50 ± 0.17	0.5 > *P* > 0.1
3	(V/3)	*Allium sativum *L.	50 ± 4.01	0.5 > *P* > 0.1	1.30 ± 0.06	0.5 > *P* > 0.1
4	(V/4)	*Althaea officinalis*L.	26 ± 5.05	*P* > 0.5	1.18 ± 0.24	*P* > 0.5
5	(V/5)	*Bauhinia variegata *L.	50 ± 12.6	0.5 > *P* > 0.1	1.36 ± 0.24	0.5 > *P* > 0.1
6	(V/6)	*Brassica nigra *(L.) W. D. J. Koch	22.5 ± 4.51	0.5 > *P* > 0.1	1.07 ± 0.11	*P* > 0.5
7	(V/7a)	*Calotropis procera *(Aiton) W. T. Aiton (exudates)	42.5 ± 2.52	0.5 > *P* > 0.1	1.30 ± 0.01	0.1 > *P* > 0.05
8	(V/7b)	*Calotropis procera *(Aiton) W. T. Aiton (flowers)	39 ± 2.02	0.5 > *P* > 0.1	1.25 ± 0.03	0.5 > *P* > 0.1
9	(V/8)	*Cedrus deodara *(Roxb. ex D. Don) G. Don	44 ± 4.04	0.5 > *P* > 0.1	1.44 ± 0.07	0.5 > *P* > 0.1
10	(V/9)	*Citrus limon *(L.) Burm. f.	39.5 ± 2.52	0.5 > *P* > 0.1	1.40 ± 0.03	0.1 > *P* > 0.05
11	(V/10)	*Citrullus colocynthis *(L.) Schrad	49.5 ± 3.53	0.5 > *P* > 0.1	1.52 ± 0.15	0.5 > *P* > 0.1
12	(V/11)	*Cuminum cyminum *L.	54 ± 1.01	0.05 > *P* > 0.02	1.48 ± 0.01	0.05 > *P* > 0.02
13	(V/12)	*Enicostemma hyssopifolium *(Willd.) I. Verd.	39.5 ± 4.54	0.5 > *P* > 0.1	1.35 ± 0.05	0.5 > *P* > 0.1
14	(V/13)	*Fagonia cretica *L.	43 ± 2.27	0.5 > *P* > 0.1	1.23 ± 0.13	*P* < 0.5
15	(V/14)	*Leucas capitata *Desf.	31.5 ± 0.50	*P* > 0.5	1.07 ± 0.05	*P* > 0.5
16	(V/15)	*Matthiola incana *(L.) W. T. Aiton	43.5 ± 3.53	0.5 > *P* > 0.1	1.44 ± 0.14	0.5 > *P* > 0.1
17	(V/16)	*Momordica charantia *L.	57 ± 5.01	0.5 > *P* > 0.1	1.5 ± 0.005	0.05 > *P* > 0.02
18	(V/17)	*Nerium indicum *Mill	47 ± 0.00	*P* < 0.05	1.24 ± 0.10	0.5 > *P* > 0.1
19	(V/18)	*Ocimum sanctum *L.	49.5 ± 7.52	0.5 > *P* > 0.1	1.5 ± 0.005	0.05 > *P* > 0.02
20	(V/19)	*Pinus roxburghii *Sarg	45 ± 1.00	*P* > 0.05	1.4 ± 0.05	0.5 > *P* > 0.1
21	(V/20)	*Pistacia integerrima *J. L. Stewart	66.5 ± 0.50	0.02 > *P* > 0.01	1.68 ± 0.10	0.5 > *P* > 0.1
22	(V/21)	*Psoralea corylifolia *L.	42.5 ± 2.50	0.5 > *P* > 0.1	1.27 ± 0.15	0.5 > *P* > 0.1
23	(V/22)	*Rhazya stricta *Decne	53.5 ± 3.53	0.5 > *P* > 0.1	1.67 ± 0.005	0.05 > *P* > 0.02
24	(V/23)	*Rubia cordifolia *L.	36 ± 1.01	0.5 > *P* > 0.1	0.93 ± 0.48	*P* > 0.5
25	(V/24)	*Sapindus mukorossi *Gaertn	44 ± 1.00	0.1 > *P* > 0.05	1.43 ± 0.12	0.5 > *P* > 0.1
26	(V/25)	*Stenolobium stans *(L.) Seem	25.5 ± 6.51	*P* > 0.5	1.1 ± 0.06	*P* > 0.5
27	(V/26)	*Terminalia arjuna *(Roxb. ex DC.) Wight and Arn	38.5 ± 1.51	0.5 > *P* > 0.1	1.27 ± 0.23	*P* > 0.5
28	(V/27)	*Trichodesma indicum *(L.) Sm	37 ± 1.00	0.5 > *P* > 0.1	0.46 ± 0.01	0.05 > *P* > 0.02
29	(V/28)	*Zingiber officinale *Roscoe	47 ± 1.00	0.05 > *P* > 0.02	1.30 ± 0.01	0.5 > *P* > 0.1
30	(V/29)	Reference standard antidote (standard antisera /immunoglobulin's)	31.5 ± 0.50	Select to compare	1.08 ± 0.02	Select to compare

**Table 5:** Cardioprotective activity of medicinal plant extracts on LDH and CK-MB levels to neutralize snake bite envenomation in experimental rabbits.

Sr. number	Group V (subgroup)	Name of evaluated sample	LDH		CK-MB	
			U/L (mean ± SEM)	*P* value/comment	U/L (mean ± SEM)	*P* value/comment
1	(V/1)	*Albizia lebbeck *(L.) Benth.	1357 ± 1.00	0.01 > *P* > 0.001	4.1 ± 0.85	0.5 > *P* > 0.1
2	(V/2)	*Allium cepa *L.	934 ± 13.03	0.1 > *P* > 0.05	14.8 ± 1.65	*P* > 0.5
3	(V/3)	*Allium sativum *L.	1177 ± 20.56	0.05 > *P* > 0.02	6.6 ± 3.30	0.5 > *P* > 0.1
4	(V/4)	*Althaea officinalis*L.	975.5 ± 33.60	0.5 > *P* > 0.1	14.8 ± 3.30	*P* > 0.5
5	(V/5)	*Bauhinia variegata *L.	1972 ± 3.00	*P* > 0.001	8.3 ± 6.76	*P* > 0.5
6	(V/6)	*Brassica nigra *(L.) W. D. J. Koch	855.5 ± 0.50	0.05 > *P* > 0.02	9.0 ± 0.85	0.5 > *P* > 0.1
7	(V/7a)	*Calotropis procera *(Aiton) W. T. Aiton (exudates)	1022 ± 5.01	0.02 > *P* > 0.01	6.6 ± 3.30	0.5 > *P* > 0.1
8	(V/7b)	*Calotropis procera *(Aiton) W. T. Aiton (flowers)	1114 ± 1.00	0.02 > *P* > 0.01	61.8 ± 10.9	0.5 > *P* > 0.1
9	(V/8)	*Cedrus deodara *(Roxb. ex D. Don) G. Don	1230 ± 23.57	0.05 > *P* > 0.02	41.2 ± 31.66	*P* > 0.5
10	(V/9)	*Citrus limon *(L.) Burm. f.	1831 ± 65.69	0.05 > *P* > 0.02	73 ± 14.1	0.5 > *P* > 0.1
11	(V/10)	*Citrullus colocynthis *(L.) Schrad	827 ± 6.51	0.1 > *P* > 0.05	05 ± 1.66	0.5 > *P* > 0.1
12	(V/11)	*Cuminum cyminum *L.	1589 ± 22.56	0.02 > *P* > 0.01	5.8 ± 0.80	0.5 > *P* > 0.1
13	(V/12)	*Enicostemma hyssopifolium *(Willd.) I. Verd.	1615 ± 1.51	*P* > 0.001	9.85 ± 1.66	0.5 > *P* > 0.1
14	(V/13)	*Fagonia cretica *L.	1418 ± 13.03	0.02 > *P* > 0.01	11 ± 2.52	*P* > 0.5
15	(V/14)	*Leucas capitata *Desf.	783 ± 10.02	0.5 > *P* > 0.1	14 ± 0.80	*P* > 0.5
16	(V/15)	*Matthiola incana *(L.) W. T. Aiton	1428 ± 6.51	*P* > 0.001	08 ± 1.66	0.5 > *P* > 0.1
17	(V/16)	*Momordica charantia *L.	1475.5 ± 3.51	*P* > 0.001	15.6 ± 2.45	*P* > 0.5
18	(V/17)	*Nerium indicum *Mill	1268 ± 12.03	0.02 > *P* > 0.01	05 ± 1.65	0.5 > *P* > 0.1
19	(V/18)	*Ocimum sanctum *L.	1335 ± 12.03	0.02 > *P* > 0.01	12.3 ± 2.45	*P* > 0.5
20	(V/19)	*Pinus roxburghii *Sarg	1050 ± 1.00	0.02 > *P* > 0.01	08 ± 1.65	0.5 > *P* > 0.1
21	(V/20)	*Pistacia integerrima *J. L. Stewart	1135.5 ± 0.5	0.02 > *P* > 0.01	13.1 ± 1.65	0.5 > *P* > 0.1
22	(V/21)	*Psoralea corylifolia *L.	1153.5 ± 0.5	*P* > 0.001	17.3 ± 2.50	0.5 > *P* > 0.1
23	(V/22)	*Rhazya stricta *Decne	1538 ± 20.05	0.02 > *P* > 0.01	4.1 ± 0.85	0.5 > *P* > 0.1
24	(V/23)	*Rubia cordifolia *L.	1078 ± 16.04	0.05 > *P* > 0.02	6.6 ± 0.00	0.5 > *P* > 0.1
25	(V/24)	*Sapindus mukorossi *Gaertn	1460.5 ± 5.51	0.01 > *P* > 0.001	4.1 ± 0.80	0.5 > *P* > 0.1
26	(V/25)	*Stenolobium stans *(L.) Seem	787 ± 28.08	*P* > 0.5	13 ± 1.76	*P* > 0.5
27	(V/26)	*Terminalia arjuna *(Roxb. ex DC.) Wight and Arn	798.5 ± 14.54	0.5 > *P* > 0.1	6.6 ± 0.00	0.5 > *P* > 0.1
28	(V/27)	*Trichodesma indicum *(L.) Sm	978.5 ± 4.51	0.05 > *P* > 0.02	6.6 ± 3.00	0.5 > *P* > 0.1
29	(V/28)	*Zingiber officinale *Roscoe	888 ± 2.00	0.05 > *P* > 0.02	17.3 ± 0.80	0.5 > *P* > 0.1
30	(V/29)	Reference standard antidote (standard antisera /immunoglobulin's)	763 ± 6.01	Select to compare	09 ± 0.85	Select to compare

## References

[1] Wuster W (1998). The cobras of the genus Naja in India. *Hamadryad*.

[2] Deraniyagala PEP (1961). The taxonomy of the cobras of south-eastern Asia, part 2. *Spolia Zeylanica*.

[3] Davidson TM, Schafer S, Killfoil J (1995). Cobras. *Wilderness and Environmental Medicine*.

[4] Asad MHHB, Razi MT, Khan T (2012). Coagulopathies in *Naja naja karachiensis* (black Pakistan cobra) bites and its effect on coagulation tests upon storage of platelet poor plasma. *Acta Poloniae Pharmaceutica*.

[5] Hider RC, Karlsson E, Namiranian S, Harvey AL (1991). Separation and purification of toxins from snake venoms. *International Encyclopedia of Pharmacology and Therapeutics 11*.

[6] Asad MHHB, Razi MT, Durr-e-Sabih (2013). Anti-venom potential of Pakistani medicinal plants: inhibition of anticoagulation activity of *Naja naja karachiensis* toxin. *Current Science*.

[7] Mebs D (2001). Toxicity in animals. Trends in evolution?. *Toxicon*.

[8] Aird SD (2002). Ophidian envenomation strategies and the role of purines. *Toxicon*.

[9] Chijiwa T, Yamaguchi Y, Ogawa T (2003). Interisland evolution of *Trimeresurus flavoviridis* venom phospholipase A_2_ isozymes. *Journal of Molecular Evolution*.

[10] Lomonte B, Angulo Y, Calderón L (2003). An overview of lysine-49 phospholipase A2 myotoxins from crotalid snake venoms and their structural determinants of myotoxic action. *Toxicon*.

[11] Dhananjaya BL, Vishwanath BS, D’Souza CJM, Mackessy SP (2009). Snake venom nucleases, nucleotidases, and phosphomonoesterases. *Handbook of Venoms and Toxins of Reptiles*.

[12] Mackessy SP, Bailey GS (1998). Phosphodiesterases, ribonucleases and deoxyribonucleases. *Enzymes from Snake Venom*.

[13] Dhananjaya BL, Nataraju A, Rajesh R (2006). Anticoagulant effect of *Naja naja* venom 5°nucleotidase: demonstration through the use of novel specific inhibitor, vanillic acid. *Toxicon*.

[14] Dhananjaya BL, D’Souza CJM (2011). The Pharmacological role of phosphatases (acid and alkaline phosphomonoesterases) in snake venoms related to release of purines—a multitoxin. *Basic and Clinical Pharmacology and Toxicology*.

[15] Girish KS, Jagadeesha DK, Rajeev KB, Kemparaju K (2002). Snake venom hyaluronidase: an evidence for isoforms and extracellular matrix degradation. *Molecular and Cellular Biochemistry*.

[16] Tan NH, Fung SY, Mackessy SP (2009). Snake venom L-amino acid oxidases. *Handbook of Venoms and Toxins of Reptiles*.

[17] Mebs D (2002). *Venomous and Poisonous Animals: A Handbook for Biologists, Toxicologists and Toxinologists, Physicians and Pharmacists*.

[18] Abdoon NA, Fatani AJ (2009). Correlation between blood pressure, cytokines and nitric oxide in conscious rabbits injected with *Leiurus quinquestriatus quinquestriatus* scorpion venom. *Toxicon*.

[19] Latimer KS, Mahaffey EA, Prasse KW (2003). *Duncan and Prasse's Veterinary Laboratory Medicine: Clinical Pathology*.

[20] Harkness JE, Turner PV, Woude SV, Wheler CL (2010). *Harkness and Wagner’s Biology and Medicine of Rabbits and Rodents*.

[21] Apple FS (1999). The specificity of biochemical markers of cardiac damage: a problem solved. *Clinical Chemistry and Laboratory Medicine*.

[22] Alkadi HO, Noman MA, Al-Thobhani AK, Al-Meklafi FS, Raja’a YA (2002). Clinical and experimental evaluation of the effect of Khat-induced myocardial infarction. *Saudi Medical Journal*.

[23] Asad MHHB, Murtaza G, Siraj S (2011). Enlisting the scientifically unnoticed medicinal plants of Pakistan as a source of novel therapeutic agents showing anti-venom activity. *African Journal of Pharmacy and Pharmacology*.

[24] Asad MHHB, Sabih DE, Chaudhary BA Anti-hemolytic (anti-venom) activity of Pakistani medicinal plants upon *Naja naja karachiensis* venom induced hemolysis.

[25] Ezenwanne EB, Ucheya RE (2012). A study of the serum concentrations of some hepatic enzymes in doses of aqueous leaf extract of *Vernonia amygdalina* in rabbits. *International Journal of Animal and Veterinary Advances*.

[26] Mirakabadi ZA, Khatoonabadi SM, Teimourzadeh SH, Sabiri GHH (2010). Serum enzymes studies in scorpion (*Hemiscorpius lepturus*) dose related envenomation in rabbits. *Archives of Razi Institut*.

[27] Green CJ, Knight J, Precious S, Simpkin S (1981). Ketamine alone and combined with diazepam or xylazine in laboratory animals: a 10 year experience. *Laboratory Animals*.

[28] Hasson SS, Al-Jabri AA, Sallam TA, Al-Balushi MS, Mothana RAA (2010). Antisnake venom activity of *Hibiscus aethiopicus* L. against *Echis ocellatus* and *Naja n. nigricollis*. *Journal of Toxicology*.

[29] Tahir Razi M, Asad MHHB, Khan T (2011). Antihaemorrhagic potentials of *Fagonia cretica* against *Naja naja karachiensis* (black Pakistan cobra) venom. *Natural Product Research*.

[30] Chethankumar M, Srinivas L (2008). Gangliosides as potential inhibitors of *Naja naja* venom PLA2 (NV-PLA2) induced human erythrocyte membrane damage. *African Journal of Biochemistry Research*.

[31] Rahmy TR, Hemmaid KZ (2000). Histological and histochemical alterations in the liver following intramuscular injection with a sublethal dose of the Egyptian cobra venom. *Journal of Natural Toxins*.

[32] Adzu B, Abubakar MS, Izebe KS, Akumka DD, Gamaniel KS (2005). Effect of *Annona senegalensis* rootbark extracts on *Naja nigricotlis nigricotlis* venom in rats. *Journal of Ethnopharmacology*.

[33] Huffman MA (2003). Animal self-medication and ethno-medicine: exploration and exploitation of the medicinal properties of plants. *Proceedings of the Nutrition Society*.

[34] Segelke BW, Nguyen D, Chee R, Xuong NH, Dennis EA (1998). Structures of two novel crystal forms of *Naja naja naja* phospholipase A_2_ lacking Ca^2+^ reveal trimeric packing. *Journal of Molecular Biology*.

[35] Lu Q, Clemetson JM, Clemetson KJ (2005). Snake venoms and hemostasis. *Journal of Thrombosis and Haemostasis*.

[36] Ehrenforth S, Schenk JF, Scharrer I (1999). Liver damage induced by coumarin anticoagulants. *Seminars in Thrombosis and Hemostasis*.

[37] Nandave M, Ojha SK, Joshi S, Kumari S, Arya DS (2007). Cardioprotective effect of *Bacopa monneira* against isoproterenol-induced myocardial necrosis in rats. *International Journal of Pharmacology*.

[38] Patel HV, Vyas AA, Vyas KA (1997). Heparin and heparan sulfate bind to snake cardiotoxin: sulfated oligosaccharides as a potential target for cardiotoxin action. *Journal of Biological Chemistry*.

[39] Yingprasertchai S, Bunyasrisawat S, Ratanabanangkoon K (2003). Hyaluronidase inhibitors (sodium cromoglycate and sodium auro-thiomalate) reduce the local tissue damage and prolong the survival time of mice injected with *Naja kaouthia* and *Calloselasma rhodostoma* venoms. *Toxicon*.

[40] Schneemann M, Cathomas R, Laidlaw ST, El Nahas AM, Theakston RDG, Warrell DA (2004). Life-threatening envenoming by the Saharan horned viper (Cerastes cerastes) causing micro-angiopathic haemolysis, coagulopathy and acute renal failure: clinical cases and review. *QJM—Monthly Journal of the Association of Physicians*.

[41] Meier J, Stocker K (1991). Effects of snake venoms on hemostasis. *Critical Reviews in Toxicology*.

[42] Marsh N, Gattullo D, Pagliaro P, Losano G (1997). The gaboon viper, *Bitis gabonica*: hemorrhagic, metabolic, cardiovascular and clinical affects of the venom. *Life Sciences*.

[43] Pipelzadeh MH, Jalali A, Taraz M, Pourabbas R, Zaremirakabadi A (2007). An epidemiological and a clinical study on scorpionism by the Iranian scorpion *Hemiscorpius lepturus*. *Toxicon*.

[44] Asad MHHB, Durr-e-Sabih, Choudary BA, Asad AF, Murtaza G, Hussain I (2014). Compensatory effects of medicinal plants of Pakistan upon prolongation of coagulation assays induced by *Naja naja karachiensis* bite. *Current Science*.

[45] Baquar SR (1989). *Medicinal and Poisonous Plants of Pakistan*.

[46] Archetti I, Tittarelli C, Cerioli M, Brivio R, Grilli G, Lavazza A Serum chemistry and hematology values in commercial rabbits, preliminary data from industrial farms in northern Italy.

